# Supplementary material for: Mitochondrial DNA copy number can influence mortality and cardiovascular disease via methylation of nuclear DNA CpGs
Source: Genome Med. 2020 Sep 28;12:84. doi: 10.1186/s13073-020-00778-7 (PMC7523322; doi:10.1186/s13073-020-00778-7)
Supplement: Supplementary file 2 — Additional file 2: Table S1. Sample characteristics of discovery and validation cohorts. Table S6. Neuroactive-ligand receptor interaction genes as identified by KEGG analysis in each approach, number of genes used in each analysis and neuroactive ligand enrichment P-value are included in brackets. Table S7. Methylation Status of Validated CpGs in TFAM KO cell lines (N = 6). Bolded entries indicate differential expression P < 0.05. Table S8. Differentially expressed genes (P < 0.05) within 1 Mb of differentially methylated CpGs in TFAM knockout cell lines. Shading indicates most differentially expressed gene for each CpG. Table S9. Results of Mendelian Randomization. A. Results for association between ARIC EA and AA derived independent cis meQTLs and mtDNA-CN. B. Results for association between ARIC meta-analysis derived independent cis meQTLs and mtDNA-CN (fixed effects model). [file 13073_2020_778_MOESM2_ESM.pdf]

**Table S1. Sample characteristics of discovery and validation cohorts.**

| | DISCOVERY COHORTS | | VALIDATION COHORTS | | | | |
|---|---|---|---|---|---|---|---|
| | ARIC AA (N=1567) | ARIC EA (N=940) | CHS | CHS AA (N=239) | CHS EA (N=294) | FHS | FHS (N=1995) |
| | *mean (range)* | *mean (range)* | | *mean (range)* | *mean (range)* | | *mean (range)* |
| **Age** | 57.2 (47 - 71) | 60.2 (47 - 72) | | 72.4 (65 - 92) | 72.3 (65 - 95) | | 61.4 (20-91) |
| **mtDNA-CN (SD units)** | 0.04 (-6.47 - 2.88) | 0.04 (-4.41 - 2.87) | | 0.05 (-3.19 - 2.69) | 0.05 (-2.28 - 2.76) | | 0.01 (-2.28 -8.11) |
| **Sex** | *N (percentage)* | *N (percentage)* | | *N (percentage)* | *N (percentage)* | | *N (percentage)* |
| Male | 604 (38.5%) | 381 (40.5%) | | 97 (41.8%) | 112 (38.9%) | | 901 (45.16%) |
| Female | 963 (61.5%) | 559 (59.5%) | | 135 (58.2%) | 176 (61.1%) | | 1094 (54.64%) |
| **Collection Site** | | | **Collection Site** | | | **Collection Site** | |
| Forsyth County, NC | 188 (12%) | 832 (88.5%) | Bowman Gray | 74 (31.9%) | 67 (23.3%) | Framingham Town | 1995 (100%) |
| Suburbs of Minneapolis, MN | 0 (0%) | 86 (9.2%) | Davis | 71 (30.6%) | 71 (24.7%) | | |
| Jackson, MS | 1379 (88%) | 0 (0%) | Hopkins | 0 (0%) | 83 (28.8%) | | |
| Washington County, MD | 0 (0%) | 22 (2.3%) | Pittsburgh | 87 (37.5%) | 67 (23.3%) | | |
| **Smoking Status** | | | | | | | |
| Current Smoker | 655 (41.8%) | 181 (19.3%) | | 32 (13.8%) | 26 (9.0%) | | 205 (10.4%) |
| Former Smoker | 484 (30.9%) | 366 (38.9%) | | 89 (38.4%) | 116 (40.3%) | | 947 (47.47%) |
| Never Smoker | 428 (27.3%) | 392 (41.7%) | | 85 (36.6%) | 133 (46.2%) | | 838 (42.0%) |
| Unknown | 0 (0.0%) | 1 (0.1%) | | 26 (11.2%) | 13 (4.5%) | | 5 (0.2%) |
| **Phenotypes (# of cases)** | | | | | | | |
| Mortality | 605 (38.6%) | 224 (23.8%) | | 194 (83.6%) | 263 (91.3%) | | 217 (10.87%) |
| ***CVD*** Prevalent | 154 (9.8%) | 49 (5.2%) | | N/A | N/A | | 94 (4.71%) |
| Incident | 296 (18.9%) | 108 (11.5%) | | 83 (35.8%) | 99 (34.4%) | | 94 (4.71%) |
| ***CHD*** Prevalent | 112 (7.1%) | 40 (4.3%) | | N/A | N/A | | 94 (4.71%) |
| Incident | 193 (12.3%) | 83 (8.8%) | | 48 (20.7%) | 57 (19.8%) | | 68 (3.41%) |
| **Cell Type Proportions** | *mean (range)* | *mean (range)* | | *mean (range)* | *mean (range)* | | *mean (range)* |
| CD8T Lymphocytes | 0.15 (0.00 - 0.48) | 0.10 (0.00 - 0.27) | | 0.09 (0.00 - 0.38) | 0.06 (0.00 - 0.22) | | 0.10 (0.00-0.36) |
| CD4T Lymphocytes | 0.19 (0.00 - 0.52) | 0.16 (0.00 - 0.44) | | 0.20 (0.00 - 0.48) | 0.15 (0.00 - 0.52) | | 0.19 (0.02-0.44) |
| B-cells | 0.07 (0.00 - 0.58) | 0.06 (0.00 - 0.56) | | 0.08 (0.00 - 0.26) | 0.06 (0.00 - 0.76) | | 0.04 (0.00-0.52) |
| Monocytes | 0.13 (0.02 - 0.26) | 0.09 (0.02 - 0.19) | | 0.10 (0.00 - 0.27) | 0.09 (0.01 - 0.35) | | 0.12 (0.05-0.30) |
| Granulocytes | 0.45 (0.15 - 0.98) | 0.55 (0.16 - 0.93) | | 0.44 (0.11 - 0.75) | 0.57 (0.03 - 0.92) | | 0.49 (0.02-0.85) |
| Natural Killer cells | N/A | 0.07 (0.00 - 0.36) | | 0.12 (0.01 - 0.38) | 0.09 (0.00 - 0.36) | | 0.02 (0.00-0.13) |

CVD: Cardiovascular disease. CHD: Coronary Heart Disease.

**Table S6. Neuroactive-ligand receptor interaction genes as identified by KEGG analysis in each approach, number of genes used in each analysis and neuroactive ligand enrichment *P*-value are included in brackets.**

| ARIC Meta-Analysis (300 CpGs, $P$=5.24x10$^{-12}$) | *TFAM* Methylation (300 CpGs, $P$=4.41x10$^{-4}$) | *TFAM* Expression (169 genes, $P$=4.30x10$^{-4}$) | *TFAM* Integrated (Methylation/Expression) (188 genes, $P$=8.77x10$^{-6}$) |
|---|---|---|---|
| *CHRM2* | *GABBR1* | *GABRB1* | *GABRB1* |
| *CHRM3* | *GRIN3B* | *MC2R* | *MC2R* |
| *CTSG* | *GABRA5* | *GH1* | *GH1* |
| *AGTR1* | *GABRB1* | *GABRA2* | *GABRA2* |
| *GABRG3* | *GABRG3* | *GABRG1* | *GABRG1* |
| *GHR* | *GABRB3* | *ADRB2* | |
| *GRIA2* | *GALR1* | *MC4R* | |
| *GRIA4* | *NPBWR1* | | |
| *P2RX1* | *GRIK1* | | |
| *P2RY2* | *GRIN2D* | | |
| *PTGER2* | *HTR1E* | | |
| *HTR1B* | *TRHR* | | |
| *NTSR1* | *TSHR* | | |
| | *VIPR2* | | |
| | *CCKBR* | | |

**Table S7. Methylation Status of Validated CpGs in *TFAM* KO cell lines (N=6).** Bolded entries indicate differential expression *P*<0.05.

| Marker Name | All Cohort Meta-Analysis | | | | Average Methylation in Negative Control Lines | Average Methylation in *TFAM* Knockout Lines | *TFAM* Differential Expression | |
|---|---|---|---|---|---|---|---|---|
| | Mean Methylation | Estimate | Standard Error | *P*-value | | | Beta Estimate | *P*-Value |
| cg03964851 (surrogate for cg21051031) | 0.83 | 0.0038 | 0.0004 | 7.34E-27 | 0.7685 | 0.7710 | -0.0025 | 9.42E-01 |
| **cg26094004** | 0.55 | -0.0079 | 0.0007 | 4.13E-28 | 0.6504 | 0.9001 | -0.2497 | 2.91E-05 |
| **cg26563141** | 0.37 | -0.0060 | 0.0008 | 2.20E-14 | 0.3071 | 0.4187 | -0.1116 | 1.25E-02 |
| cg14575356 | 0.55 | 0.0033 | 0.0005 | 1.22E-09 | 0.7906 | 0.7918 | -0.0013 | 9.40E-01 |
| cg23513930 | 0.35 | 0.0020 | 0.0003 | 3.71E-09 | Not on EPIC array and no surrogate available | | | |
| **cg08899667** | 0.58 | -0.0041 | 0.0006 | 1.55E-12 | 0.7931 | 0.7014 | 0.0917 | 3.33E-03 |

***Note:** Mean methylation for cg21051031 = 0.85

**Table S8. Differentially expressed genes (*P*<0.05) within 1 Mb of differentially methylated CpGs in *TFAM* knockout cell lines. Shading indicates most differentially expressed gene for each CpG.**

| EWAS CpG | Chr: Position | Number of Genes Within 1 Mb | *P*-Value for *TFAM* Methylation Difference | Gene | Test Statistic for *TFAM* Expression | *P*-value for *TFAM* Expression | Direction of Effect (following KO)* | Distance from CpG (Kb) | Description |
|---|---|---|---|---|---|---|---|---|---|
| **cg26094004** | **17: 42,075,116** | **42** | 2.91E-05 | *ACLY* | 4.9871 | 2.55E-02 | Negative | 144.6 | ATP citrate lyase [Source:HGNC Symbol;Acc:HGNC:115] |
| | | | | *KAT2A* | 5.0487 | 2.46E-02 | Negative | 38.0 | lysine acetyltransferase 2A [Source:HGNC Symbol;Acc:HGNC:4201] |
| | | | | *HSPB9* | 8.3116 | 3.94E-03 | Negative | 46.3 | heat shock protein family B (small) member 9 [Source:HGNC Symbol;Acc:HGNC:30589] |
| | | | | *KCNH4* | 9.5817 | 1.97E-03 | Negative | 81.8 | potassium voltage-gated channel subfamily H member 4 [Source:HGNC Symbol;Acc:HGNC:6253] |
| | | | | *COASY* | 4.3154 | 3.78E-02 | Negative | 486.4 | Coenzyme A synthase [Source:HGNC Symbol;Acc:HGNC:29932] |
| | | | | *CCR10* | 4.5647 | 3.26E-02 | Positive | 603.8 | C-C motif chemokine receptor 10 [Source:HGNC Symbol;Acc:HGNC:4474] |
| | | | | *RAMP2* | 11.9339 | 5.51E-04 | Positive | 683.3 | receptor activity modifying protein 2 [Source:HGNC Symbol;Acc:HGNC:9844] |
| | | | | *AOC3* | 4.2112 | 4.02E-02 | Positive | 776.1 | copper containing 3 [Source:HGNC Symbol;Acc:HGNC:550] |
| | | | | *IFI35* | 16.9886 | 3.76E-05 | Positive | 931.6 | interferon induced protein 35 [Source:HGNC Symbol;Acc:HGNC:5399] |
| | | | | *RND2* | 8.9365 | 2.80E-03 | Positive | 950.1 | Rho family GTPase 2 [Source:HGNC Symbol;Acc:HGNC:18315] |
| | | | | *BRCA1* | 4.0986 | 4.29E-02 | Positive | 969.2 | DNA repair associated [Source:HGNC Symbol;Acc:HGNC:1100] |
| **cg26563141** | **2: 88,124,876** | **4** | 1.25E-02 | *RPIA* | 20.8215 | 5.04E-06 | Negative | 566.8 | ribose 5-phosphate isomerase A [Source:HGNC Symbol;Acc:HGNC:10297] |
| **cg08899667** | **6: 31,761,055** | **32** | 3.33E-03 | *CCHCR1* | 8.0190 | 4.63E-03 | Positive | 602.8 | coiled-coil alpha-helical rod protein 1 [Source:HGNC Symbol;Acc:HGNC:13930] |
| | | | | *HLA-C* | 4.6442 | 3.12E-02 | Positive | 488.9 | major histocompatibility complex: class I: C [Source:HGNC Symbol;Acc:HGNC:4933] |
| | | | | *HLA-B* | 9.4318 | 2.13E-03 | Positive | 403.9 | major histocompatibility complex: class I: B [Source:HGNC Symbol;Acc:HGNC:4932] |
| | | | | *ATP6V1G2* | 7.1309 | 7.58E-03 | Positive | 212.6 | ATPase H+ transporting V1 subunit G2 [Source:HGNC Symbol;Acc:HGNC:862] |
| | | | | *NFKBIL1* | 5.4633 | 1.94E-02 | Positive | 202.2 | NFKB inhibitor like 1 [Source:HGNC Symbol;Acc:HGNC:7800] |
| | | | | *CLIC1* | 4.2279 | 3.98E-02 | Positive | 21.3 | chloride intracellular channel 1 [Source:HGNC Symbol;Acc:HGNC:2062] |
| | | | | *MSH5* | 13.4158 | 2.50E-04 | Positive | 1.8 | mutS homolog 5 [Source:HGNC Symbol;Acc:HGNC:7328] |
| | | | | *HSPA1L* | 4.7247 | 2.97E-02 | Positive | 48.6 | heat shock protein family A (Hsp70) member 1 like [Source:HGNC Symbol;Acc:HGNC:5234] |
| | | | | *C2* | 5.9338 | 1.49E-02 | Positive | 136.7 | complement C2 [Source:HGNC Symbol;Acc:HGNC:1248] |
| | | | | *FKBPL* | 8.1388 | 4.33E-03 | Positive | 367.7 | FK506 binding protein like [Source:HGNC Symbol;Acc:HGNC:13949] |
| | | | | *EGFL8* | 4.5363 | 3.32E-02 | Negative | 403.5 | EGF like domain multiple 8 [Source:HGNC Symbol;Acc:HGNC:13944] |
| | | | | *HLA-DRA* | 10.6612 | 1.09E-03 | Positive | 678.8 | major histocompatibility complex: class II: DR alpha [Source:HGNC Symbol;Acc:HGNC:4947] |
| | | | | *HLA-DRB5* | 24.7584 | 6.50E-07 | Negative | 756.3 | major histocompatibility complex: class II: DR beta 5 [Source:HGNC Symbol;Acc:HGNC:4953] |
| | | | | *HLA-DRB1* | 4.1996 | 4.04E-02 | Positive | 817.7 | major histocompatibility complex: class II: DR beta 1 [Source:HGNC Symbol;Acc:HGNC:4948] |

*Positive beta indicates that after the decrease in mtDNA-CN (*TFAM* Knockout), expression has increased as compared to controls.

**Table S9. Results of Mendelian Randomization. A.** Results for association between ARIC EA and AA derived independent cis meQTLs and mtDNA-CN. **B.** Results for association between ARIC meta-analysis derived independent cis meQTLs and mtDNA-CN (fixed effects model).

**A.**

| ARIC Cohort | Chr | meQTL CpG from EWAS | CpG Position | meQTL SNP | SNP Position | MAF | Imputation Quality ($R^2$) | mtDNA~meQTL SNP | | | CpG~meQTL SNP | | | Power for MR |
|---|---|---|---|---|---|---|---|---|---|---|---|---|---|---|
| | | | | | | | | Beta Estimate | Standard Error | *P*-value | Beta Estimate | Standard Error | *P*-value | |
| **EA (Permuted *P*=7.84E-04)** | 17 | cg26094004 | 42,075,116 | rs11654132 | 42,149,134 | 0.178 | 0.97 | -0.0362 | 0.0842 | 0.67 | 0.0322 | 0.0043 | 8.80E-14 | 0.99 |
| | 6 | cg08899667 | 31,761,055 | rs3117574 | 31,725,230 | 0.098 | 1 | 0.0362 | 0.0762 | 0.64 | 0.0149 | 0.0031 | 1.76E-06 | 0.29 |
| | 6 | cg08899667 | 31,761,055 | rs9267653 | 31,840,415 | 0.305 | 0.97 | -0.0127 | 0.0547 | 0.82 | 0.0101 | 0.0022 | 6.42E-06 | 0.28 |
| | 5 | cg21051031 | 93,905,482 | rs2973154 | 93,930,488 | 0.195 | 0.97 | 0.0112 | 0.0664 | 0.87 | 0.0073 | 0.0021 | 4.47E-04 | 0.75 |
| **AA (Permuted *P*=9.12E-04)** | 3 | cg23513930 | 10,334,717 | rs154236 | 10,356,314 | 0.254 | 0.96 | -0.0692 | 0.0416 | 0.10 | -0.0064 | 0.0009 | 2.23E-11 | 0.86 |
| | 6 | cg08899667 | 31,761,055 | rs28366163 | 31,704,804 | 0.148 | 0.94 | 0.0244 | 0.0474 | 0.61 | 0.0097 | 0.0019 | 4.64E-07 | 0.75 |
| | 6 | cg14575356 | 130,013,903 | rs1894642 | 130,015,331 | 0.41 | 1 | 0.0358 | 0.0353 | 0.31 | -0.0056 | 0.0012 | 3.95E-06 | 0.58 |
| | 6 | cg14575356 | 130,013,903 | rs17469966 | 130,061,044 | 0.057 | 0.82 | 0.1076 | 0.0867 | 0.21 | -0.0129 | 0.003 | 1.70E-05 | 0.49 |
| | 6 | cg08899667 | 31,761,055 | rs9267659 | 31,846,234 | 0.057 | 0.98 | 0.1007 | 0.0875 | 0.25 | 0.0151 | 0.0036 | 2.52E-05 | 0.56 |
| | 6 | cg14575356 | 130,013,903 | rs9398917 | 130,014,226 | 0.238 | 0.98 | -0.0150 | 0.0393 | 0.70 | -0.0046 | 0.0014 | 6.44E-04 | 0.34 |

**B.**

| Chr | meQTL CpG from EWAS | CpG Position | meQTL SNP | SNP Position | Analysis | MAF | Imputation Quality ($R^2$) | mtDNA~meQTL SNP | | | CpG~meQTL SNP | | | Power for MR |
|---|---|---|---|---|---|---|---|---|---|---|---|---|---|---|
| | | | | | | | | Beta Estimate | Standard Error | *P*-value | Beta Estimate | Standard Error | *P*-value | |
| 6 | cg08899667 | 31,761,055 | rs9267653 | 31,840,415 | Meta | | | -0.0156 | 0.0318 | 0.62 | 0.0086 | 0.0013 | 1.99E-11 | |
| | | | | | EA | 0.305 | 0.97 | -0.0127 | 0.0547 | 0.82 | 0.0101 | 0.0022 | 6.42E-06 | 0.28 |
| | | | | | AA | 0.246 | 0.97 | -0.0154 | 0.0389 | 0.69 | 0.0078 | 0.0015 | 5.31E-07 | 0.75 |
| 6 | cg08899667 | 31,761,055 | rs28366163 | 31,704,804 | Meta | | | 0.0347 | 0.0428 | 0.42 | 0.0107 | 0.0017 | 1.10E-09 | |
| | | | | | EA | 0.063 | 0.96 | 0.1318 | 0.1033 | 0.20 | 0.0143 | 0.0041 | 4.54E-04 | 0.18 |
| | | | | | AA | 0.148 | 0.94 | 0.0244 | 0.0474 | 0.61 | 0.0096 | 0.0019 | 4.18E-07 | 0.75 |
